# Robotic Assisted Radical Cystectomy with Extracorporeal Urinary Diversion Does Not Show a Benefit over Open Radical Cystectomy: A Systematic Review and Meta-Analysis of Randomised Controlled Trials

**DOI:** 10.1371/journal.pone.0166221

**Published:** 2016-11-07

**Authors:** Wei Shen Tan, Pramit Khetrapal, Wei Phin Tan, Simon Rodney, Marisa Chau, John D. Kelly

**Affiliations:** 1 Division of Surgery and Interventional Science, University College London, London, United Kingdom; 2 Department of Urology, University College London Hospital, London, United Kingdom; 3 Department of Urology, Rush University Medical Center, Chicago, Illinois, United States of America; Eberhard Karls University, GERMANY

## Abstract

**Background:**

The number of robotic assisted radical cystectomy (RARC) procedures is increasing despite the lack of Level I evidence showing any advantages over open radical cystectomy (ORC). However, several systematic reviews with meta-analyses including non-randomised studies, suggest an overall benefit for RARC compared to ORC. We performed a systematic review with meta-analysis of randomised controlled trials (RCTs) to evaluate the perioperative morbidity and efficacy of RARC compared to ORC in patients with bladder cancer.

**Methods:**

Literature searches of Medline/Pubmed, Embase, Web of Science and clinicaltrials.gov databases up to 10^th^ March 2016 were performed. The inclusion criteria for eligible studies were RCTs which compared perioperative outcomes of ORC and RARC for bladder cancer. Primary objective was perioperative and histopathological outcomes of RARC versus ORC while the secondary objective was quality of life assessment (QoL), oncological outcomes and cost analysis.

**Results:**

Four RCTs (from 5 articles) met the inclusion criteria, with a total of 239 patients all with extracorporeal urinary diversion. Patient demographics and clinical characteristics of RARC and ORC patients were evenly matched. There was no significant difference between groups in perioperative morbidity, length of stay, positive surgical margin, lymph node yield and positive lymph node status. RARC group had significantly lower estimated blood loss (p<0.001) and wound complications (p = 0.03) but required significantly longer operating time (p<0.001). QoL was not measured uniformly across trials and cost analysis was reported in one RCTs. A test for heterogeneity did highlight differences across operating time of trials suggesting that surgeon experience may influence outcomes.

**Conclusions:**

This study does not provide evidence to support a benefit for RARC compared to ORC. These results may not have inference for RARC with intracorporeal urinary diversion. Well-designed trials with appropriate endpoints conducted by equally experienced ORC and RARC surgeons will be needed to address this.

## Introduction

Radical cystectomy and lymphadenectomy remains the recommended curative treatment for muscle invasive bladder cancer and recurrent high grade non-muscle invasive bladder cancer [1]. In recent years, robotic assisted radical cystectomy (RARC) has become the surgical approach of choice in a number of high volume institutions [2–4].

Minimally invasive surgery seeks to reduce post-operative morbidity and allow an early return to normal activity while replicating the principles of open surgery and maintaining oncological equivalence [5]. The benefits of minimally invasive surgery in colorectal cancer is supported by level one evidence. Patients who had laparoscopic colorectal cancer resections had similar oncological outcomes, enhanced postoperative recovery, shorter hospital length of stay (LOS) and lower use of parenteral narcotics with a similar post-operative complications, mortality and hospital readmission rates [6].

Previous systematic reviews with meta-analyses were conducted to determine the benefits for RARC, and concluded that patients undergoing RARC have a lower post-operative morbidity, a shorter LOS and higher lymph node yield compared to open radical cystectomy (ORC) [7–10]. However, these reviews incorporated retrospective and prospective cohort studies which are subject to significant bias. Furthermore, two more RCTs have since been published after these four reviews, and the addition of these studies may aid in determining the benefits of RARC over ORC. To date, there has been no systematic review with meta-analysis which includes data exclusively from RCTs of RARC versus open radical cystectomy (ORC).

Therefore, the primary objective of this systematic review is to compare RARC versus ORC on perioperative and histopathological outcomes. Secondary outcomes include quality of life assessment, oncological outcomes and cost analysis.

## Methods

### Search strategy and study selection

A systemic search of the literature was performed in MEDLINE/PubMed, Embase, Web of Science and clinictrials.gov databases up till 10^th^ March 2016. The following keywords and MeSH terms were used: (bladder cancer OR transitional cell carcinoma OR urothelial cell carcinoma OR urinary bladder cancer OR urinary bladder neoplasm OR urinary bladder tumor OR urinary bladder tumour OR urinary bladder carcinoma) AND (cystectomy OR cystoprostatectomy OR bladder resection) AND (robotic OR da vinci OR robotic-assisted OR robotic assisted) AND (open) AND (randomised OR randomized). Only studies published in English were included. All conference abstracts, review articles, editorials, comments, letters to the editor and duplicate records were excluded.

**Fig 1:**
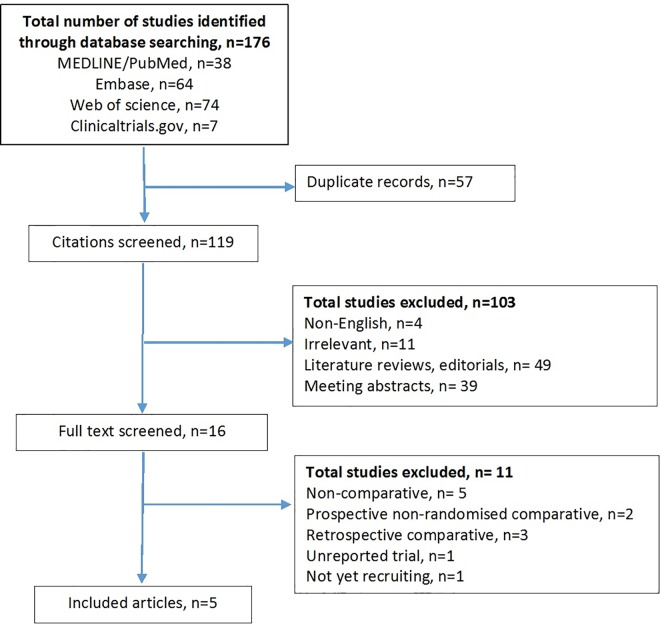
Flow chart of studies identified, excluded and included.

The inclusion criteria for eligible studies were: 1) RCTs and 2) comparisons between ORC and RARC for bladder cancer. The exclusion criteria were: 1) non-English studies and 2) conference abstracts, literature reviews, editorials, comments, and letters to the editor. Abstracts and full text articles for eligible studies were independently screened by two authors. When there was a discrepancy, the study was discussed with a third author. The PRISMA flowchart and checklist is shown in Fig 1 and S1 Table respectively. Risk of bias for each study was assessed by two authors independently using the Cochrane ‘risk of bias table’.

### Data extraction and outcome of interest

The following data were extracted from studies which met the inclusion criteria:

#### Patient demographics

Age, gender, body mass index (BMI), American Society of Anesthesiologists (ASA) score, type of urinary diversion, pathological T staging, previous pelvic or abdominal surgery and use of neo-adjuvant chemotherapy (NAC).

#### Perioperative variables

Estimated blood loss (EBL), blood transfusion requirement, operative time, length of hospital stay (LOS), quality of life (QoL) assessment and 90-day postoperative complications. Complications were classified according to the modified Memorial Sloan-Kettering Cancer Center (MSKCC) Clavien-Dindo (CD) system [11]. Minor and major complications were defined as CD I-II and CD III-IV respectively.

#### Oncological variables

Cystectomy histopathological tumour and nodal stage (according to 2002 TNM classification) [12], positive surgical margins (PSM), mean lymph node yield and positive lymph node status.

### Statistical analysis

The meta-analysis was conducted using Review Manager software v.5.3 (Cochrane Collaboration, Oxford, UK). The weighted mean difference (WMD) and odds ratio (OR) were used to compare continuous and dichotomous variables respectively. For studies presenting continuous data as median and range or interquartile range (IQR), mean and standard deviation was calculated according to methodology described by Hozo et al. [13].

Study heterogeneity was assessed for each outcome using Cochrane’s χ^2^ test, with p<0.10 indicating evidence of heterogeneity. Degree of heterogeneity was quantified using the I^2^ statistic, with I^2^ ≥25% indicating substantial heterogeneity. A random-effect model was used to attempt to account for significant heterogeneity. Statistical significance was set at p<0.05 in all tests.

A sensitivity analysis was performed using R v3.2.4 (Lucent Technologies, New Jersey, USA) using the metafor package. A ‘leave one out’ algorithm was used to assess the influence of each individual study. The meta-analysis was also repeated using risk ratios as the outcome statistic instead of odds ratio.

## Results

### Characterisation of eligible studies

One-hundred and seventy-six citations were identified from the database search (Fig 1). After screening of citations, 16 full text studies were reviewed and six manuscripts from five RCTs were met the inclusion criteria [14–19]. No published data was available for one RCT which closed early due to poor recruitment [19]. The remaining four RCTs contributed to 239 patients (RARC: 121, ORC: 118). Four RCTs reported perioperative complications [14–17], three studies reported QoL data [15, 17, 18], one study reported oncological outcomes [15] and one performed cost analysis [17]. One of the four studies had a third group treated with laparoscopic cystectomy and this group was not included in the analysis [15]. A full risk of bias assessment is shown in a ‘risk of bias table’ in S2 Table.

### Patient demographics and clinical characteristics

Patient demographics and clinical characteristics are shown in Tables 1 and 2. There was no baseline difference for RARC and ORC patients in age, sex, BMI, ASA and T-stage in all four studies. Three studies excluded patients with extensive previous abdominal surgery and one study did not specify this [16]. Similarly, data from three studies reported no difference in NAC use and data was not available in one study [16].

**Table 1:** Characteristics of included studies.

First author and reference	Recruitment	Country	Primary end point	Number of patients, ORC/ RARC	Male sex, ORC/ RARC	Age, median/ mean, ORC/ RARC	IC patients, ORC/ RARC	NB patients, ORC/ RARC	Match factors
Nix et al. 2010 [16]	April 2008- Jan 2009	USA	Lymph node yield	20/ 21	17/ 14	69.2/ 67.4	14/ 14	6/ 7	1,2,3,4,7,8
Parekh et al. 2013 [14]	July 2009- June 2011	USA	Feasibility study	20/ 20	16/ 18	64.5/ 69.5	NA	NA	1,2,3,4,5,6, 7
Bochner et al. 2015 [17]	March 2010- March 2013	USA	Perioperative complication	58/ 60	42/ 51	65.0/ 66.0	23/ 27	35/ 33	1,2,3,4,5,6,7,8
Khan et al. 2016 [15]	March 2009- July 2012	UK	Perioperative outcomes	20/ 20	18/ 15	66.6/ 68.6	17/ 18	3/ 2	1,2,3,4,5,6,7,8

1 = age, 2 = gender, 3 = BMI, 4 = ASA, 5 = previous abdominal surgery, 6 = neoadjuvant chemotherapy, 7 = clinical stage, 8 = diversion type, ORC: open radical cystectomy, RARC: robotic assisted radical cystectomy, IC: ileal conduit, NB: neobladder

**Table 2:** Analysis of patient demographics and clinical variables comparing RARC vs ORC.

	Number of RARC/ ORC patients	WMD/ OR (95% CI)	P value	X^2^	Study heterogeneity		
					df	I^2^ (%)	P value
Age	121/ 118	1.14 [-0.70, 3.61]	0.19	2.82	3	0	0.42
Proportion of males	121/118	1.15 [0.61, 2.14]	0.67	6.51	3	54%	0.09
BMI	100/ 98	-0.65 [-2.01, 0.70]	0.34	0.54	2	0	0.76
ASA I-II	36/31	1.46 [0.65, 3.30]	0.36	0.08	1	0	0.78
ASA III-IV	44/47	0.68 [0.30, 1.54]	0.36	0.08	1	0	0.78
Previous NAC	100/98	1.22 [0.63, 2.34]	0.56	0.81	2	0	0.67
Pathological T stage: ≤pT2	85/85	0.75 [0.38, 1.49]	0.41	1.24	3	0	0.74
Pathological T stage: ≥pT3	36/33	1.36 [0.67, 2.75]	0.40	1.20	3	0	0.75

BMI: body mass index, ASA: American Society of Anesthetics, NAC: neoadjuvant chemotherapy, ORC: open radical cystectomy, RARC: robotic assisted radical cystectomy, WMD: weighted mean difference, OR: odds ratio, CI: confidence interval

Urinary diversion from the robotic group of all four RCTs were performed by an extracorporeal approach. More patients underwent ileal conduit urinary diversion (113 patients vs 86 patients) compared to neobladder, even though there were a similar number of neobladders were reconstructed between RARC and ORC groups (RARC: 42, ORC: 44). One study did not report type of urinary diversion constructed [14].

### Perioperative outcomes

#### Intraoperative outcomes: Estimated blood loss, blood transfusion rate and requirement and operating time

**Fig 2:**
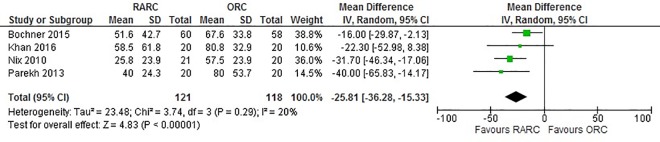
Forest plot and meta-analysis of blood loss (10ml).

**Fig 3:**
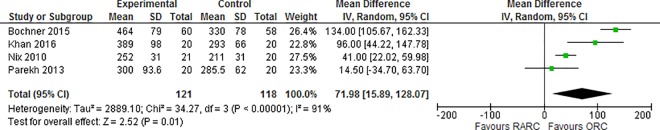
Forest plot and meta-analysis of operating time (mins).

Pooling data from 239 patients showed that EBL was significantly lower in RARC group compared to ORC (p<0.0001) (Fig 2). Only one RCT with 40 cases, reported blood transfusion rate and requirements and showed no significant difference in both median units of blood transfused (RARC: 0 vs ORC: 2, p = 0.410) and requirements (RARC: 8/20 vs ORC: 10/20, p = 0.410) [14]. Pooled data from all four studies suggested that RARC was associated with significantly longer operative times (WMD: 71.98 mins; 95% CI (15.89, 128.07); p = 0.01) (Fig 3).

#### Postoperative complications: Length of stay, 90-day all complications, 90-day major complications, 90-day mortality and complication type

**Fig 4:**
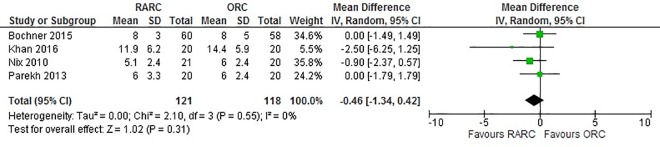
Forest plot and meta-analysis of length of stay.

**Fig 5:**
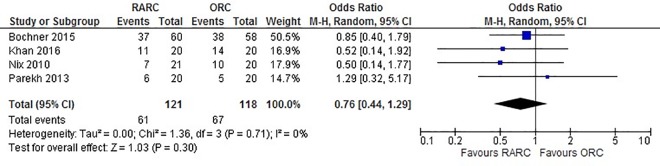
Forest plot and meta-analysis of all complications.

**Fig 6:**
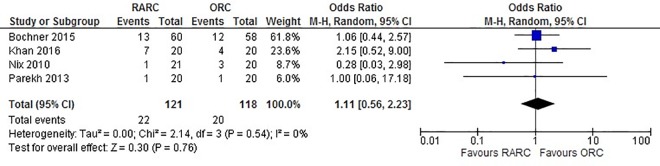
Forest plot and meta-analysis of major complications.

Data extracted from all four studies did not show a significant difference between LOS when RARC was compared to ORC (WMD: -0.46 days; 95% CI (-1.34, 0.42); p = 0.30) (Fig 4). Pooled data from 239 patients did not show a difference in all 90-day complications in the RARC and ORC groups (OR: 0.75; 95% CI (0.44, 1.28); p = 0.29) (Fig 5). Similarly, no significant difference was observed in 90-day major complications between both groups (OR: 1.11; 95% CI (0.55, 2.22); p = 0.77) (Fig 6). No difference was observed in 90-day mortality between RARC and ORC (OR: 0.32; 95% CI (0.03, 3.00); p = 0.32). Wound complication was the only complication which was significantly lower in RARC compared to ORC (OR: 0.23; 95% CI (0.03, 0.88); p = 0.03) (Table 3).

**Table 3:** Analysis of perioperative complications according to Memorial classification.

Complications	Number of RARC/ ORC patients	WMD/ OR (95% CI)	P value	X_2_	Study heterogeneity		
					df	I^2^ (%)	P value
Bleeding	121/ 118	1.27 (0.30, 5.29)	0.75	0.41	1	0	
Cardiac	121/ 118	1.06 [0.48, 2.32]	0.88	0.99	3	0	0.80
Gastrointestinal	121/ 118	0.66 [0.40, 1.10]	0.11	1.34	3	0	0.72
Genitourinary	121/ 118	0.81 [0.27, 2.45]	0.71	4.92	3	39	0.18
Infectious	121/ 118	1.18 [0.80, 1.73]	0.40	0.80	3	0	0.85
Miscellaneous	121/ 118	0.55 [0.12, 2.52]	0.44	0.15	1	0	0.70
Neurologic	121/ 118	1.38 [0.42, 4.58]	0.60	2.30	3	0	0.51
Pulmonary	121/ 118	0.32 [0.03, 3.01]	0.32	N/A	N/A	N/A	N/A
Surgical	121/ 118	1.40 [0.23, 8.64]	0.72	1.22	2	0	0.54
Thromboembolic	121/ 118	1.24 [0.43, 3.52]	0.69	0.75	2	0	0.69
Wound	121/ 118	0.23 [0.06, 0.88]	0.03	0.02	1	0	0.89
Death	121/ 118	0.32 [0.03, 3.00]	0.32	0.00	1	0	1.00

RARC: robotic assisted radical cystectomy, ORC: open radical cystectomy, WMD: weighted mean difference, OR: odds ratio, CI: confidence interval, NA: not applicable

#### Histopathological variables: Positive surgical margin (PSM), lymph node count and positive lymph node status

**Fig 7:**
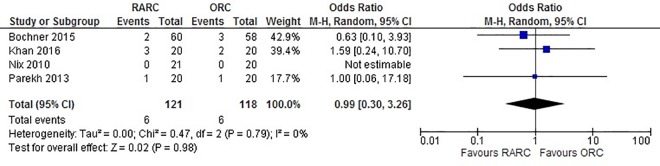
Forest plot and meta-analysis of positive surgical margin.

**Fig 8:**
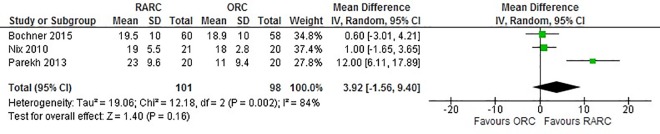
Forest plot and meta-analysis of lymph node yield.

**Fig 9:**
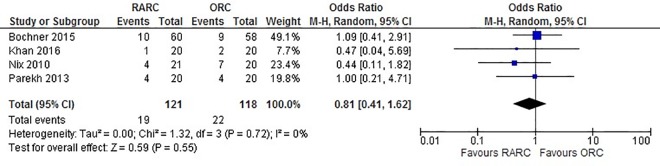
Forest plot and meta-analysis of lymph node positive status.

Data from four studies that accessed PSM status showed no significant difference between the RARC and ORC groups (OR: 0.98; 95% CI (0.29, 3.23); p = 0.97) (Fig 7). There was also no significant difference between lymph node yield (WMD: 3.89; 95% CI (-1.55, 9.33); p = 0.16) (Fig 8) and positive lymph node status (WMD: 0.84; 95% CI (0.48, 1.47); p = 0.54) (Fig 9) between RARC and ORC groups. Bochner et al. was the only study to divide lymph node dissection (LND) to standard and extended [17]. While only lymph node yield of standard dissection was used for meta-analysis to avoid introducing heterogeneity in the analysis, no difference in lymph node yield between RARC and ORC was observed in an extended LND (p = 0.5).

### Quality of life outcomes

Although three studies evaluated the QoL postoperatively, different questionnaires were used, hence pooled analysis of data was not possible [15, 17, 18]. Messer et al. used the Functional Assessment of Cancer Therapy–Vanderbilt Cystectomy which were completed 3-monthly for 12 months [18], Bochner et al. used the European Organisation for Research and Treatment of Cancer (EORTC) QLQ-C30 questionnaire which was completed at 3 and 6 months postoperatively [17], while Khan et al. used the Functional Assessment of Cancer Therapy-General, Functional Assessment of Cancer Therapy-Bladder and Trial Outcome Index questionnaire which was completed at a mean of 8 months postoperatively [15]. However, all studies concluded that there was no significant difference in QoL between the RARC and ORC groups.

### Oncological outcomes

Of the four studies, only one study reported oncological outcomes with no significant difference in recurrence free survival (RFS) (RARC: 73.6%; ORC: 89.0%; p = 0.5), cancer specific survival (CCS) (RARC: 100%; ORC: 100%; p = 1.0) and overall survival (OS) (RARC: 95%; ORC: 100%; p = 0.1) [15].

### Cost analysis

Only one study performed cost analysis based on Medicare reimbursement [17]. Patients who had RARC with neobladder reconstruction generated an average additional average cost of $3,920 compared to ORC patients (*p* < 0.0001) whereas patients who had an ileal conduit following RARC incurred an additional average cost of $1,740 compared to ORC (*p* < 0.05). Longer operating time attributed to 98% and 69% of additional cost in ileal conduit and neobladder patients respectively.

### Heterogeneity of studies

Significant heterogeneity was detected between studies in lymph node yield and operating time. This is likely attributed to differences in surgical technique and experience between surgeons. Analysing pooled data using the random-effect model was performed to reduce the effect of between-study heterogeneity.

### Sensitivity analysis

The ‘leave-one-out’ sensitivity analysis demonstrated that none of the conclusions would change if any one study was removed for each outcome variable. The full results of this are contained in the S1–S8 Figs. Repeating the summary estimates using risk ratio instead of odds ratio for the dichotomous variables; all complications, major complications, positive surgical margins and lymph node status, again revealed no change to the findings.

## Discussion

This is the first systematic review and meta-analysis of randomised controlled trials comparing the outcomes of RARC and ORC. Previously, there have been four other systematic reviews on this topic, however these included both retrospective and prospective comparative studies which were at high risk of selection, reporting and publication bias [7, 8, 10, 20]. These meta-analyses have concluded that RARC is associated with lower perioperative complications, reduced LOS, higher lymph node yield, lower transfusion requirement and equivocal PSM. The current meta-analysis comprising of pooled data with 239 patients from four RCTs does not support the conclusions from non-RCT meta-analysis [7, 8, 10, 20]. The results of the current meta-analysis show that RARC is associated with lower EBL, lower wound complications rate and longer operating times. However, no significant difference is observed in 90-day perioperative complications, LOS, lymph node yield, PSM and QoL. A sensitivity analysis demonstrating that neither choice of statistical outcome measure nor any individual RCT impacted on the results supports the validity of the conclusions in this report.

Comparisons between morbidity rates reported for individual surgical series is often challenging due to significant variation in surgical technique, prior operative experience and documentation of complications [21]. 90-day complication rates of between 30% and 77% have been reported for RARC with extracorporeal urinary diversion [22]. To standardise reporting methodology for radical cystectomy, a modified Clavien-Dindo classification has been proposed [11]. All RCTs used either traditional Clavien-Dindo or modified classification system to standardise reporting.

In this analysis, we did not find a significant difference in 90-day perioperative complications between studies. A recent study analysed complications following RARC with intracorporeal urinary diversion in 134 cases and found that the majority of Clavien ≥III complications can be attributed to a surgical cause which may be related to surgeon experience [23]. In our meta-analysis of operating time, there was significant heterogeneity observed which may reflect a variation in surgical experience in RARC. None of the RCTs reported prior surgical experience for either RARC or ORC, and therefore it was difficult to determine this. Although the learning curve to achieve minimal perioperative complications is yet to be defined, a minimum of 30 cases is suggested to achieve adequate lymph node yield and PSM [24] while experience of more than 100 cases has been put forward as a minimum to be considered very experienced [25]. In robotic assisted laparoscopic prostatectomy (RALP), perioperative complications continue to improve and plateau after 150 cases while improvements in urinary incontinence and sexual function outcomes were observed until after 600 cases [26, 27]. Hence, these results may not be as heterogeneous if RARC was performed by experienced surgeons.

Patients undergoing radical cystectomy are often older, smoke tobacco and have co-morbidities such as cardiovascular and renal dysfunction, making them susceptible to perioperative complications. A single arm study in RARC with intracorporeal urinary diversion reported that poor cardiorespiratory fitness measured by cardiopulmonary exercise testing did not predict 30-day perioperative complications [28]. In colon cancer, a large RCT of minimally invasive versus open colectomy did not show differences in 60-day complications but did report significantly shorter LOS (p≤0.001) and lower use of opiate based analgesia (p≤0.001) [6]. Hence, it has been hypothesised that RARC will reduce perioperative morbidity or at the very least shorten LOS compared to ORC which is contrary to our findings. While there is no RCT comparing RALP with open radical prostatectomy (ORP), RALP has now succeeded ORP as the most common surgical approach for radical prostatectomy with excellent perioperative outcomes [29]. In comparison to previous meta-analyses, the current review did not show a reduction in LOS between RARC and ORC.

Urinary diversion reconstruction, accounts for the majority of complications following radical cystectomy [30]. All previous systematic review and meta-analyses included in this meta-analysis performed urinary diversion reconstruction using an extracorporeal approach. The requirement for a mini laparotomy for the urinary diversion reconstruction has been postulated to negate potential perioperative benefits of a minimally invasive approach and with intracorporeal urinary diversion gaining popularity, the question remains whether the approach to diversion reconstruction will have an impact on perioperative outcomes.

All previous systematic reviews and meta-analyses including our current review consistently report that RARC is associated with a significantly lower EBL translating to a lower blood transfusion rate. This could be attributed to a more precise and controlled dissection using the robotic platform as well as pneumoperitoneum. No RCT has been designed to measure the effects of perioperative transfusion on either functional recovery or oncological outcome in cystectomy. Evidence that blood transfusion is associated with increased 30-day morbidity and mortality stems from the analysis of 10,100 patients who had non-cardiac surgery [31]. In radical cystectomy, a study of 1,490 consecutive cases showed that perioperative blood transfusion was associated with increased cancer specific mortality and overall mortality [32]. These small but highly significant effects may require a large sample size to uncover which would be very difficult to prove in a RCT and to alter practice would be based on inference.

PSM and lymph node yield are indicators of surgical quality. The presence of soft tissue PSM in particular reduces 5-year cancer specific survival to 32% (95% CI: 19–54) from 72% (95% CI: 69–75) [33]. In an analysis of 4,410 ORC patients with the overall incidence of a PSM was 6.3%, PSM was associated with higher pathological T stage; PSM for pT1, pT2, pT3 and pT4 was 1.8%, 2.3%, 7.6% and 24.0% respectively [34]. This meta-analysis shows no significant difference in PSM between RARC and ORC however only 18.0% of patients in the meta-analysis were ≥pT3 disease. In a series of 184 ORC and RARC cases, no difference in PSM have been reported between RARC and ORC [2].

Retrospective studies have shown that a higher lymph node yield of at least 8 is associated with cancer specific survival even in node negative disease [35]. Comparing lymph node yield is confounded by factors such as the use of NAC, pathological stage of disease, surgeon and method of pathological evaluation. None of the RCTs included an adjustment for case mix and the meta-analysis did not show a difference in lymph node yield between RARC and ORC. The Southwest Oncology Group (SWOG) S1011 (NCT01224665) trial is still ongoing and will address the issue if extended LND is necessary. Three of the four RCTs performed a standard template while Bochner et al. used both standard and extended LND with comparable lymph node yield suggesting that the quality of LND in RARC is equivocal to ORC [17].

It was not possible to pool QoL data for this analysis as QoL was assessed by different tools and at different time points. Among the three RCTs to date, there has been no difference in QoL reported for RARC compared to ORC. In the colorectal literature, patients treated with laparoscopic surgery showed better QoL in the early postoperative phase but this was no longer evident in longer term follow up [36]. However, a recent RCT comparing open retropubic prostatectomy with robotic assisted radical prostatectomy failed to show any significant difference between early functional outcomes as well as quality of life measured at 12 weeks postoperatively [37]. All three RCTs assessed QoL between 3–8 months post-surgery. It is possible that any potential gain from a minimally invasive approach may have been undetected. A further limitation will be the sample size for individual studies. A health economic analysis has not been conducted by any of the RCTs however, one study did perform a cost analysis and attributed higher cost for RARC to longer operating time [17].

Limitations of this systematic review with meta-analysis include the small sample size for pooled data. In addition, each of the RCTs were conducted at a single institution. This is evident in operating time heterogeneity and might reflect individual surgeon experience rather than surgical technique. To date all RCTs have either been feasibility studies, have closed before planned recruitment or were designed to measure surrogate endpoints. The pooled data set comprised 239 cases in total, and the systematic review with meta-analysis was not conducted on individual patient data and a test for heterogeneity has highlighted that surgical experience may have influenced the results. A further consideration is the conversion from a truly minimally invasive approach to open surgery for urinary diversion reconstruction which could confound the benefits of minimally invasive surgery.

## Conclusion

This study is the first systematic review with meta-analysis to include data from only RCTs of ORC versus RARC. Unlike previous systematic reviews with meta-analyses, which have included observational data, our results do not show a benefit for RARC compared to ORC. There are significant issues with the trials which have been conducted in RARC which may influence the outcome and integrity of the meta-analysis at this time. RARC with intracorporeal urinary diversion remains an evolving technique and high quality RCTs will be required to determine benefit. In addition, RCTs should be performed by equally experienced ORC and RARC surgeons. For the present, the role of RARC and whether the technique can challenge ORC as the standard of care remains unanswered.

## Supporting Information

S1 Fig Leave one out sensitivity analysis for outcome blood loss (10 ml). (TIF)

S2 Fig Leave one out sensitivity analysis for outcome operating time. (TIF)

S3 Fig Leave one out sensitivity analysis for outcome length of stay. (TIF)

S4 Fig Leave one out sensitivity analysis for all complications. (TIF)

S5 Fig Leave one out sensitivity analysis for all major complications. (TIF)

S6 Fig Leave one out sensitivity analysis for positive surgical margins. (TIF)

S7 Fig Leave one out sensitivity analysis for outcome lymph node yield. (TIF)

S8 Fig Leave one out sensitivity analysis for lymph node positive status. (TIF)

S1 Table PRISMA Checklist. (DOC)

S2 Table Risk of bias assessment. (DOCX)
