# Supplementary figures and images for: Robotic Assisted Radical Cystectomy with Extracorporeal Urinary Diversion Does Not Show a Benefit over Open Radical Cystectomy: A Systematic Review and Meta-Analysis of Randomised Controlled Trials
Source: PLoS One. 2016 Nov 7;11(11):e0166221. doi: 10.1371/journal.pone.0166221 (PMC5098822; doi:10.1371/journal.pone.0166221)

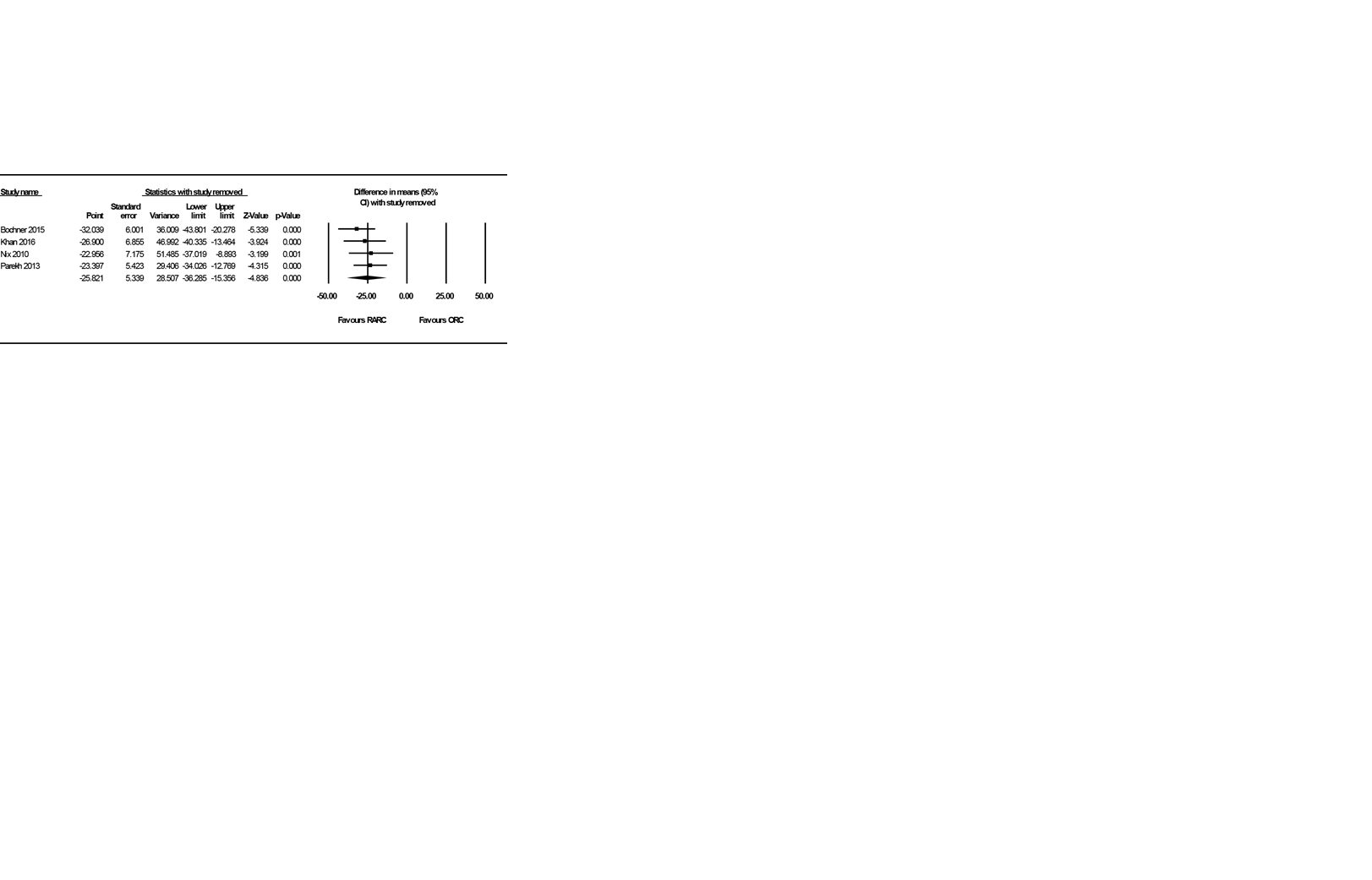

Supplement: S1 Fig — (TIF) [file pone.0166221.s001.tif]

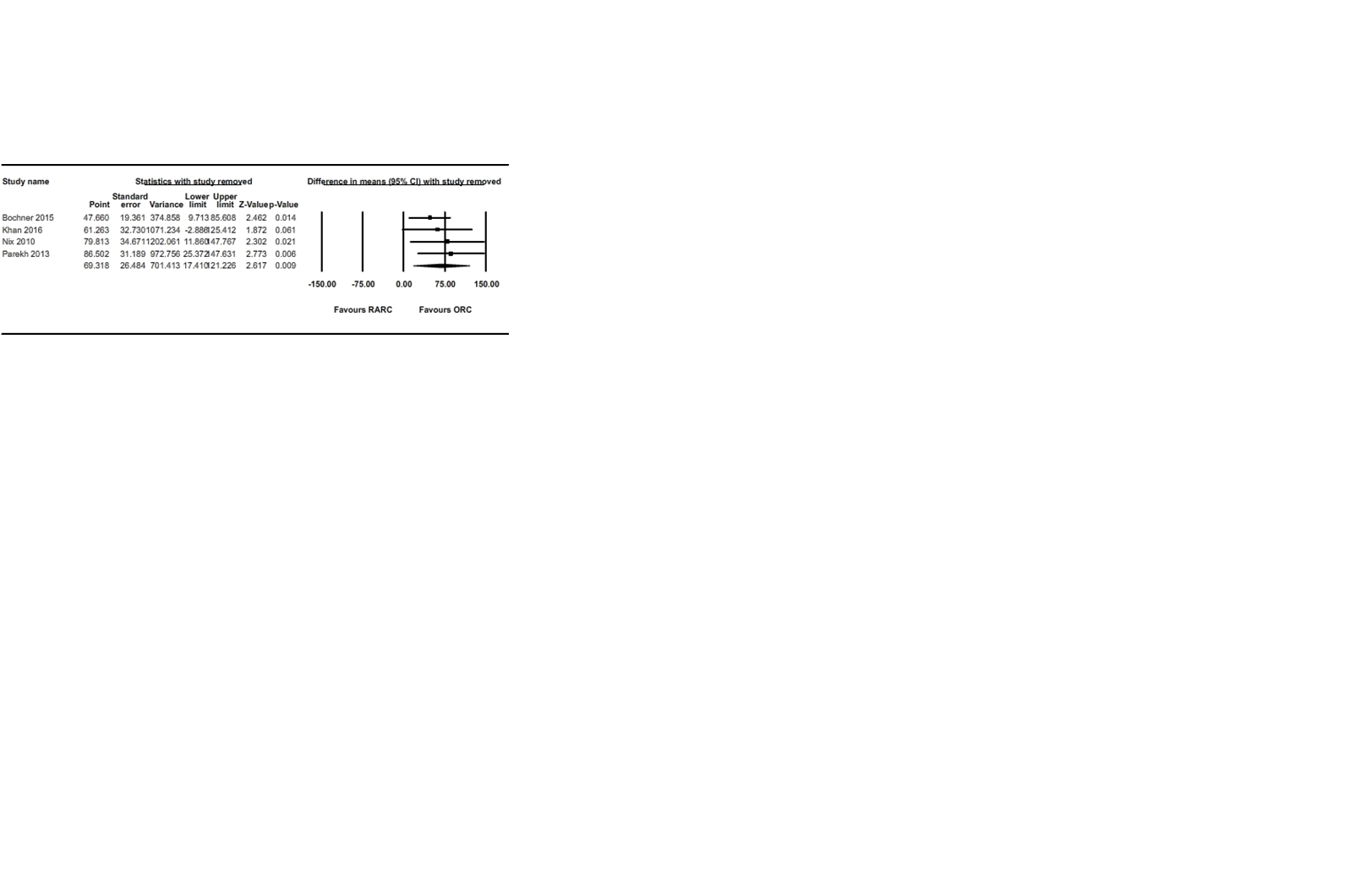

Supplement: S2 Fig — (TIF) [file pone.0166221.s002.tif]

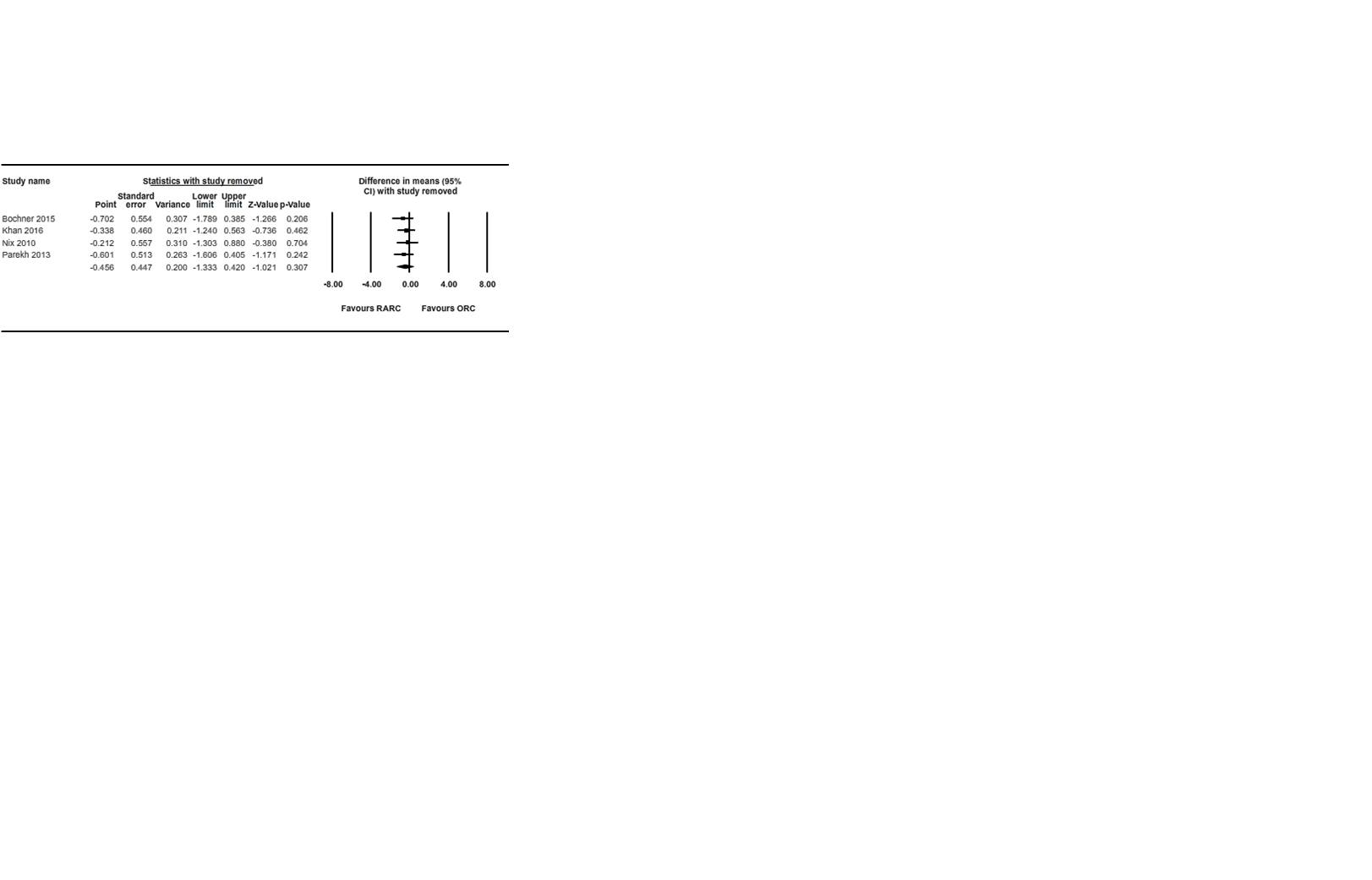

Supplement: S3 Fig — (TIF) [file pone.0166221.s003.tif]

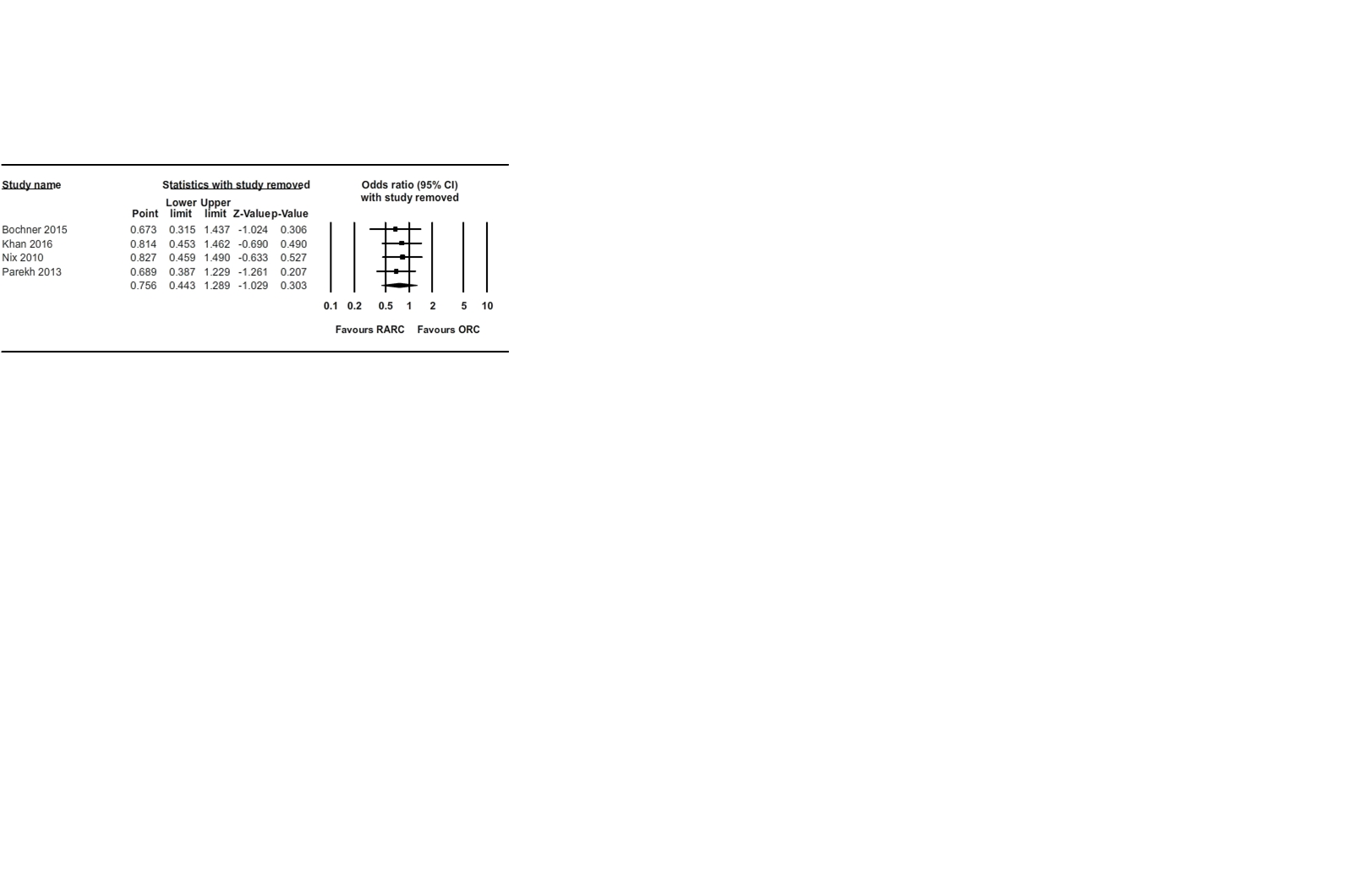

Supplement: S4 Fig — (TIF) [file pone.0166221.s004.tif]

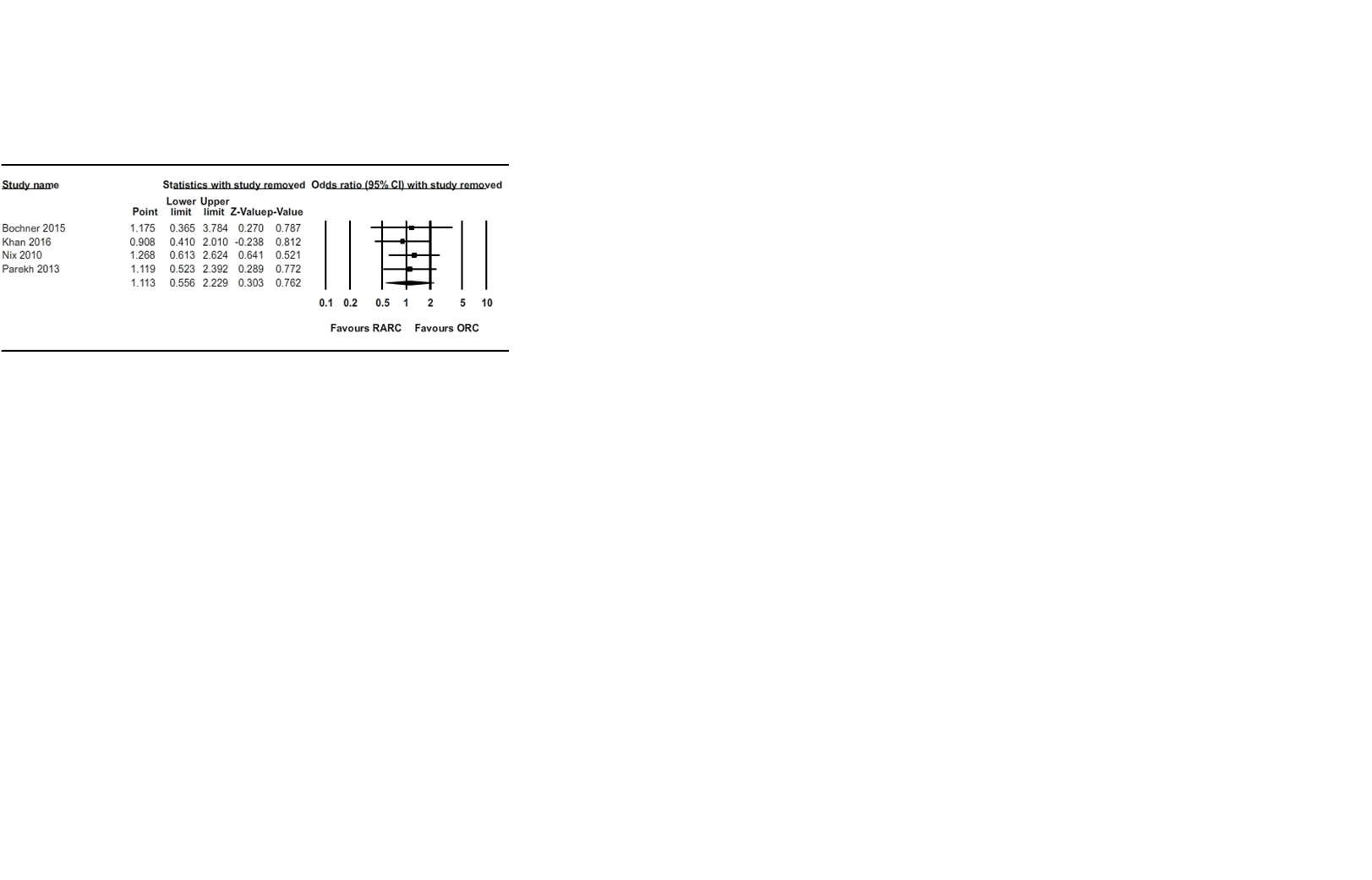

Supplement: S5 Fig — (TIF) [file pone.0166221.s005.tif]

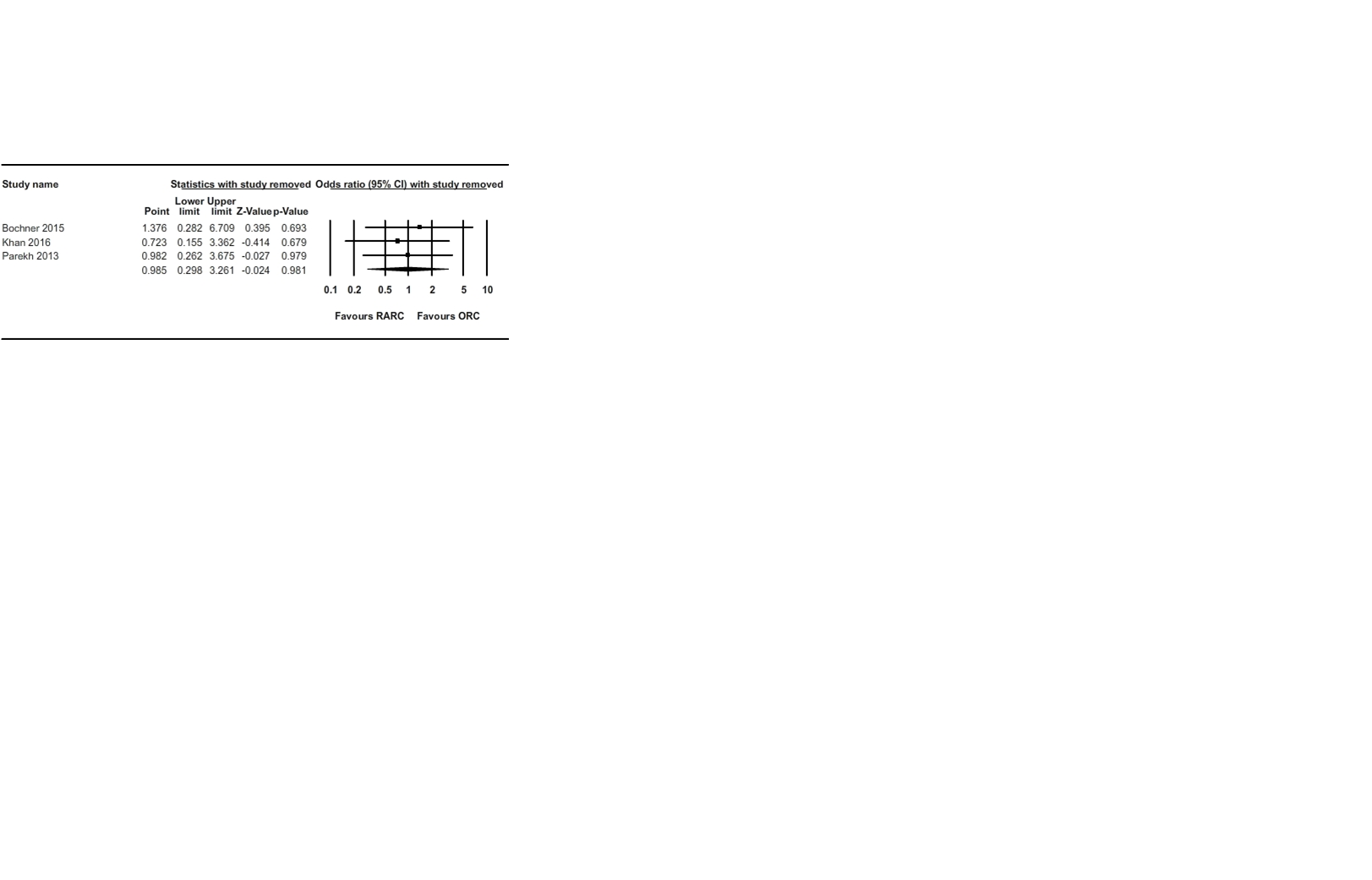

Supplement: S6 Fig — (TIF) [file pone.0166221.s006.tif]

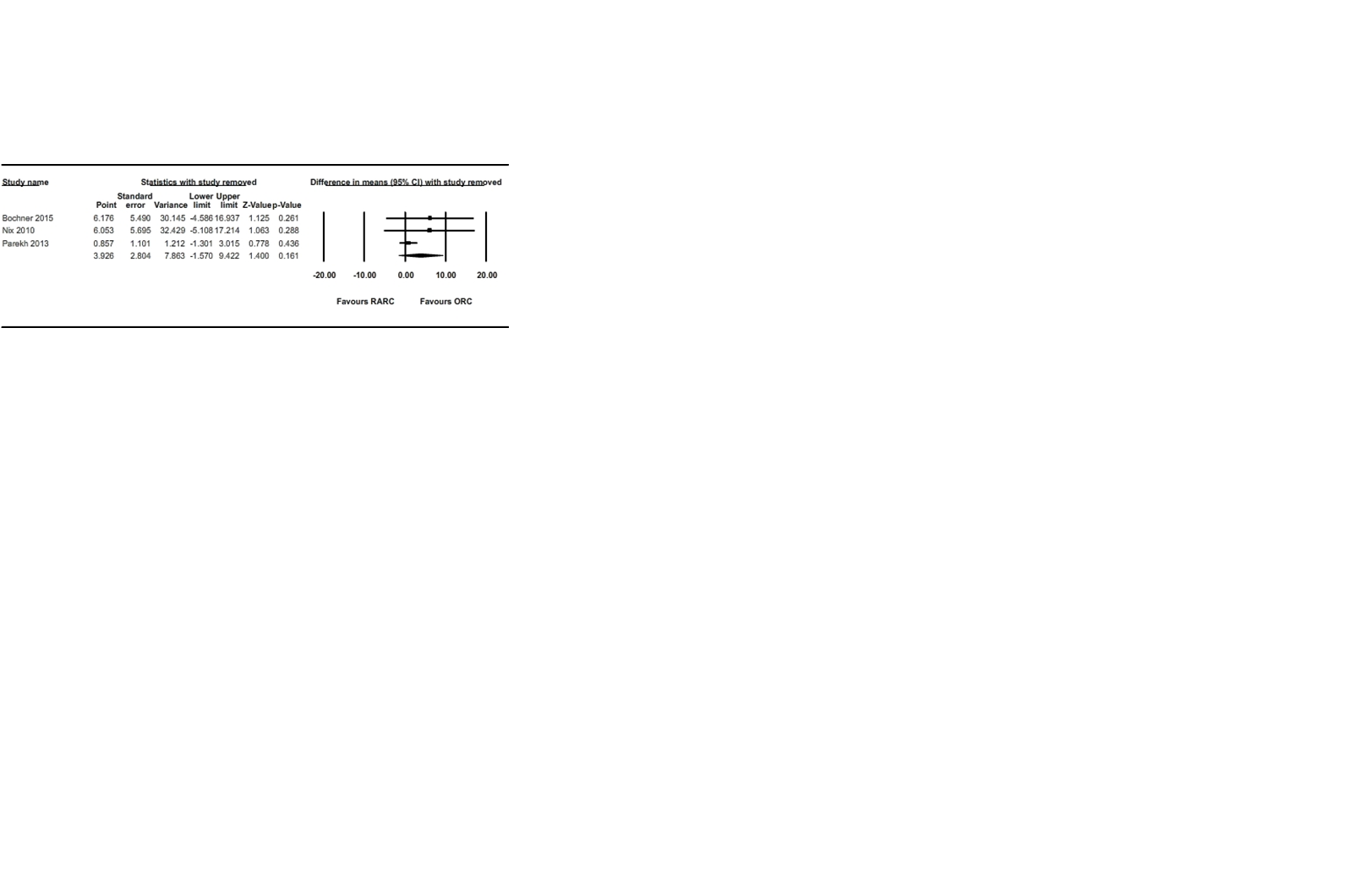

Supplement: S7 Fig — (TIF) [file pone.0166221.s007.tif]

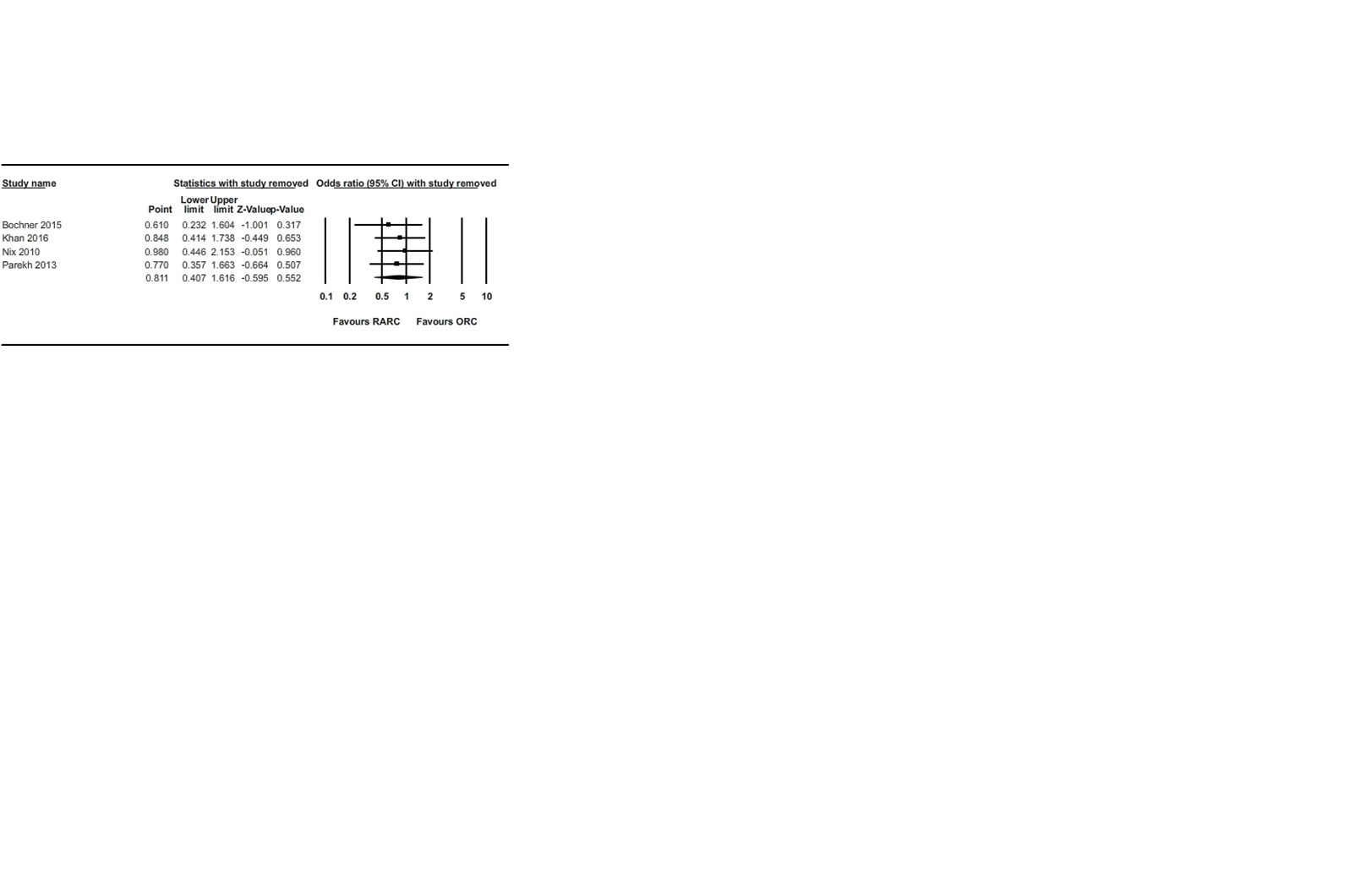

Supplement: S8 Fig — (TIF) [file pone.0166221.s008.tif]
