# Supplementary material for: Robotic Assisted Radical Cystectomy with Extracorporeal Urinary Diversion Does Not Show a Benefit over Open Radical Cystectomy: A Systematic Review and Meta-Analysis of Randomised Controlled Trials
Source: PLoS One. 2016 Nov 7;11(11):e0166221. doi: 10.1371/journal.pone.0166221 (PMC5098822; doi:10.1371/journal.pone.0166221)
Supplement: S2 Table — (DOCX) [file pone.0166221.s010.docx]

# Supporting Information

S2 Table: Risk of bias assessment

| **Author** | **Bias** | **Author’s judgment** | **Support for judgment** |
| --- | --- | --- | --- |
| Bochner et al. | Random sequence generation (selection bias) | Low risk | Patients were stratified by age (≤64 vs ≥64 yr) and American Society of Anaesthesiologist score (1-2 vs 3-4), then randomly assigned 1:1 using randomly permuted blocks of random length. |
|  | Allocation concealment (selection bias) | Low risk | Randomisation performed by independent office where allocation concealment was ensured by a password-protected database |
|  | Blinding of participants and researchers (performance bias) | High risk | Blinding not possible |
|  | Blinding of outcome assessment (detection bias) | High risk | Blinding not possible |
|  | Incomplete outcome data (attrition bias) | Low risk | No patients lost to follow-up |
|  | Selective reporting (reporting bias) | Low risk | All pre-specified outcomes were reported |
|  | Other bias | Low risk | None |
| Nix et al. | Random sequence generation (selection bias) | High risk | The randomisation schema was performed with five sequential patients undergoing an approach before alternating surgical modality |
|  | Allocation concealment (selection bias) | High risk | Randomisation schema of five sequential patients may allow the investigator to predict allocation concealment |
|  | Blinding of participants and researchers (performance bias) | High risk | Blinding not possible |
|  | Blinding of outcome assessment (detection bias) | High risk | Low risk |
|  | Incomplete outcome data (attrition bias) | Low risk | No patients lost to follow-up |
|  | Selective reporting (reporting bias) | Low risk | All pre-specified outcomes were reported |
|  | Other bias | Low risk | None |
| Parekh et al. | Random sequence generation (selection bias) | Low risk | Computerised randomisation |
|  | Allocation concealment (selection bias) | Low risk | Each assignment was placed in a sealed envelope with the corresponding slot number written on the outside. At the time of consent, the lowest numbered envelope remaining was opened and the patient was assigned to the surgical procedure listed on the piece of paper inside the envelope. |
|  | Blinding of participants and researchers (performance bias) | High risk | Blinding not possible |
|  | Blinding of outcome assessment (detection bias) | High risk | Blinding not possible |
|  | Incomplete outcome data (attrition bias) | Low risk | No patients were lost to follow-up |
|  | Selective reporting (reporting bias) | Low risk | Peri-operative pathological outcomes were not reported for one patient. Reasons for missing outcome data unlikely to result in bias |
|  | Other bias | Low risk | None |
| Khan et al. | Random sequence generation (selection bias) | Low risk | Simple randomisation |
|  | Allocation concealment (selection bias) | Low risk | Allocation envelopes were opened by the patient in the presence of three members of the research team to ensure that no changes were made to allocation |
|  | Blinding of participants and researchers (performance bias) | High risk | Blinding not possible |
|  | Blinding of outcome assessment (detection bias) | High risk | Blinding not possible |
|  | Incomplete outcome data (attrition bias) | Low risk | One patient lost to follow-up. Reasons for missing outcome data unlikely to be related to true outcome |
|  | Selective reporting (reporting bias) | Low risk | All pre-specified outcomes were reported |
|  | Other bias | Low risk | None |
